# Development of a Two-Finger Haptic Robotic Hand with Novel Stiffness Detection and Impedance Control

**DOI:** 10.3390/s24082585

**Published:** 2024-04-18

**Authors:** Vahid Mohammadi, Ramin Shahbad, Mojtaba Hosseini, Mohammad Hossein Gholampour, Saeed Shiry Ghidary, Farshid Najafi, Ahad Behboodi

**Affiliations:** 1 Department of Biomechanics, University of Nebraska Omaha, Omaha, NE 68106, USA; vmohammadi@unomaha.edu (V.M.); raminshahbad@unomaha.edu (R.S.); 2 Institute of Computer Science, University of Bonn, 53115 Bonn, Germany; 3 Mechanical and Aerospace Engineering Department, Old Dominion University, Norfolk, VA 23508, USA; mghol004@odu.edu; 4 Center for Health Innovation, Staffordshire University, Staffordshire ST4 2XE, UK; saeed.shiryghidary@staffs.ac.uk; 5 School of Mechatronic System Engineering, Simon Fraser University, Surrey, BC V5A 1S6, Canada

**Keywords:** robotics, haptic, robotic hand, impedance control, grasp, gripper

## Abstract

Haptic hands and grippers, designed to enable skillful object manipulation, are pivotal for high-precision interaction with environments. These technologies are particularly vital in fields such as minimally invasive surgery, where they enhance surgical accuracy and tactile feedback: in the development of advanced prosthetic limbs, offering users improved functionality and a more natural sense of touch, and within industrial automation and manufacturing, they contribute to more efficient, safe, and flexible production processes. This paper presents the development of a two-finger robotic hand that employs simple yet precise strategies to manipulate objects without damaging or dropping them. Our innovative approach fused force-sensitive resistor (FSR) sensors with the average current of servomotors to enhance both the speed and accuracy of grasping. Therefore, we aim to create a grasping mechanism that is more dexterous than grippers and less complex than robotic hands. To achieve this goal, we designed a two-finger robotic hand with two degrees of freedom on each finger; an FSR was integrated into each fingertip to enable object categorization and the detection of the initial contact. Subsequently, servomotor currents were monitored continuously to implement impedance control and maintain the grasp of objects in a wide range of stiffness. The proposed hand categorized objects’ stiffness upon initial contact and exerted accurate force by fusing FSR and the motor currents. An experimental test was conducted using a Yale–CMU–Berkeley (YCB) object set consisted of a foam ball, an empty soda can, an apple, a glass cup, a plastic cup, and a small milk packet. The robotic hand successfully picked up these objects from a table and sat them down without inflicting any damage or dropping them midway. Our results represent a significant step forward in developing haptic robotic hands with advanced object perception and manipulation capabilities.

## 1. Introduction

Haptics refers to the sense of touch and tactile sensations and serves as the term for technology that enables these touch-based interactions. In the context of robot design and control, haptic technology specifically encompasses interactions between humans and machines and between machines and objects [1]. This technology is dedicated to creating and advancing touch-based devices and machines that mimic the capabilities of the human hand for grasping and manipulating objects [2]. During object–machine interactions, the device will encounter various sensory feedback experiences from objects, including pressure and temperature perception, surface texture recognition, vibrations, stiffness, and force feedback. Consequently, multiple feedback from the object will allow the machine to explore a three-dimensional perception based on the object’s size, weight, and surface characteristics. Haptics allows users to interact with machines by transmitting and conveying motor control action [3]. This information, resembling the afferent sensory information in the human nervous system, enables haptics to apply controlled forces to objects and simulate the sense of manipulation for the operator. Given the frequent and crucial requirement to skillfully grasp objects, haptic hands and grippers are extensively employed in crucial subfields where precise object control and smooth interaction with the environment are crucial. Notably, in various contexts, such as minimally invasive surgeries [4,5], advanced prosthetic limbs, and industrial automation and manufacturing [6], the central importance of haptic hands becomes evident. Given the significance of these applications, the design and enhancement of these hands carry paramount importance. Through continuous improvement, these hands empower robots to execute delicate tasks precisely and replicate human touch sensations. Thus, a multitude of researchers in the field of robotics have directed their efforts toward advancing the kinematics and control mechanisms of haptic hands and grippers, all in pursuit of enhancing object manipulation capabilities. Their specific emphasis is on skillfully navigating the uncertainties associated with unknown object characteristics and their dynamic interactions with the robot. Various control strategies, including tactile, visual, and force feedback, have been individually employed or integrated in tandem to mitigate these uncertainties.

An end effector holds a central role within haptic robots as a pivotal component for orchestrating interactions between the robot and its working environment. It contains tasks ranging from smoothly grasping to skillfully manipulating objects. Consequently, a robot’s performance is closely intertwined with the efficacy of its end effector’s design. End effectors manifest in diverse configurations, yet hands and grippers emerge as predominant components in the context of haptic robots. Grippers, characterized by a straightforward design with, typically, two jaws, facilitate uncomplicated actions such as direct grasping [7,8,9]. On the other hand, robotic hands are more intricate, often featuring articulated fingers with additional degrees of freedom that mimic human-like bending and flexing. This heightened dexterity and precision position robotic hands to excel in tasks demanding a refined touch and an intricate maneuvering field [10,11]. This work aims to develop a highly versatile robotic hand capable of effectively grasping and manipulating objects that exhibit a wide range of stiffness levels. We evaluated the ability of this hand, equipped with five electronic servomotors, to grasp a diverse range of unknown objects, each varying in stiffness and weight. The key inputs for the proposed manipulation process include force data, servomotor currents, and kinematic information. 

The structure of this paper is as follows: Section 2 presents the background and approaches used for designing this device. Section 3 introduces detailed information about the prototype and controller design of the haptic hand. Section 4 provides the experimental implementation details, including experiment setup and protocols, followed by the preliminary experimental results in Section 5. Finally, Section 6 concludes this paper.

## 2. Background and Approach

Numerous researchers have put forth various grippers and robotic hands, each designed to manipulate diverse objects. Despite the diversity in approaches, the unifying goal is to ensure robotic systems can manipulate objects seamlessly, avoiding any potential damage or unintended impact by integrating sophisticated control algorithms and innovative design features. 

In a pioneering study in 1989, Mark Cutkosky developed a haptic gripper model with two arms, each possessing different degrees of freedom, to explore the implementation of touch capabilities for human perception and to study the applicability of human body mechanisms to enhance the design of robotic systems [12]. A collaborative venture between the National Aeronautics and Space Administration (NASA) and the Defense Advanced Research Projects Agency (DARPA) resulted in the development of a tactile glove designed for their dexterous humanoid robot [13]. This study delved into the utilization of diverse sensory data and the iterative evolution of the glove’s design. The findings underscored the significant enhancements observed in the robot’s grasping proficiency and its adeptness in tool manipulation when using force and tactile data. The results demonstrated the promising impact of this integrated approach on augmenting the robot’s capabilities for intricate tasks and complex interactions. A sophisticated tactile feedback system incorporating force sensors, a control system, and a pneumatic balloon tactile display was developed and implemented in the DaVinci surgical robot [14]. The successful integration of this system resulted in a notable reduction in the force applied by surgeons during surgeries. Leveraging haptic feedback, the technology optimized the application of force, thereby enhancing the precision and delicacy with which tissues were handled during surgical procedures. After conducting experiments with 20 individuals of varying ages and skill levels, they found that, on average, each person could employ optimal force after five minutes of working with this tool. Ultimately, the group found that the average force consumption in the presence of the tactile feedback system was 4.6 Newtons less than the condition without the tactile feedback system.

Dollar and Howe introduced a two-finger grasper with dual degrees of freedom on each finger [15,16]. Their study delved into joint-coupling schemes for grasping in unstructured environments. This research assessed the performance of a simple, underactuated gripper in diverse conditions, focusing on its efficacy in grasping objects with minimal contact forces. The study leveraged motion equations to implement grasping and highlighted the impact of the stiffness ratio and kinematic configuration on the grasper’s ability to manipulate objects, demonstrating its adaptability even without prior information about object location and size. In 2009, Romano et al. introduced an innovative grasp approach, drawing inspiration from human mechanisms, to enhance the performance of a parallel-jaw gripper mounted on a PR2 robotic arm [17]. The PR2 was equipped with a precision gear mechanism, enabling accurate position control. Complementing this, a force controller enabled the PR2 to achieve high-accuracy loading interactions with objects. Through the integration of haptic sensory feedback with a fine position and a force-controlling algorithm, this grasping controller significantly elevated the robot’s proficiency in safely and securely lifting a diverse array of objects from a table, effectively mitigating the risks of damage or slippage. 

In a separate study, Bicchi et al. designed a gripper to apply the appropriate force to objects with varying stiffness [18]. Their approach involved utilizing feedback from both the displacement of objects and force sensors. This combined sensory information was employed to determine the stiffness of objects, enabling the gripper to apply the requisite force tailored to the specific stiffness characteristics of each object. In 2018, Montana and Suarez introduced a novel gripping approach centered on enhancing the grasp quality of unknown objects [19]. Their focus involved refining hand configuration, grasp quality, and object positioning, relying solely on tactile and kinematic information gathered during object manipulation. This approach aimed to optimize the manipulation of unfamiliar objects through a strategic combination of tactile feedback and kinematic adjustments.

While a predominant reliance on tactile feedback is evident in many studies, a subset of investigations has underscored the significance of force feedback as a central element in their approaches. This shift toward prioritizing force data suggests an evolving trend in the field, indicating that force-based methodologies offer unique insights and advantages for addressing the challenges associated with manipulating unknown objects. This approach, as demonstrated by Shaw and Dubey, enabled the application of optimized forces, leading to the swift and effective handling of flexible and delicate objects while minimizing slippage [20]. Instead of tactile sensors, they employed data from flexible FlexiForce sensors at each fingertip as the input, with servo angles serving as the output for adjusting the appropriate loading. Control over the appropriate servo angles was achieved through a proportional–integral–derivative (PID) controller, operating based on an innovative anti-slip algorithm. This methodology showcased a departure from traditional tactile-based systems, highlighting the effectiveness of force-centric approaches in enhancing the manipulation of objects with varying properties. In a separate study, M. Al-Mohammed et al. devised an adaptive closed-loop grasping algorithm capable of applying minimal force to grip objects without causing damage or slippage [21]. This back-stepping adaptive algorithm utilized gripper velocity as the controller input and effectively measured slip velocity and force for detecting instances of slippage. This approach demonstrated an advanced control strategy to achieve secure and damage-free grasping during object manipulation.

These studies have presented a variety of algorithms and techniques geared toward enhancing gripper capabilities in object manipulation without causing damage. However, these approaches are not without limitations. For instance, the gripper employed in [17] lacked the precision required to accurately grasp asymmetrical objects due to its parallel jaws. The mechanism designed by [18] is primarily aimed at measuring the stiffness of various objects, lacking sufficient accuracy for other applications. Furthermore, the gripper developed by [15,16], utilizing compliant actuators, demonstrated lower force capabilities. Lastly, the gripper designed in [20] exhibited limitations in dexterity. Given these constraints, the present study seeks to address these shortcomings by proposing a new gripper model. Additionally, drawing inspiration from the methodologies presented in these studies, our research endeavors to introduce a novel controlling algorithm that builds upon the insights gained from the limitations of existing approaches.

Our objective was to design a robotic hand using inexpensive sensors and feedback capable of gripping unknown objects. To this end, our proposed hand operated reactively, executing localized control over movements and contact forces to safeguard against object drop and breakage. Our design followed an iterative approach, with force and kinematic configurations serving as inputs for each iteration. Forces on each fingertip were accurately computed using force sensors to initiate a preliminary sense of touch and acquire essential insights into the unknown object’s geometry. Moreover, the average current values from each servomotor were employed to discern object stiffness, facilitating object categorization based on this attribute. Current data were transformed into an impedance form and harnessed as the primary input for a control algorithm. This algorithm, in turn, optimized the grip and lifting of each object, ensuring slip-free and breakage-free handling. The inverse kinematic measurements played a vital role as a control observer, dynamically adjusting the geometric positions of fingers and joints to ensure precise grasping and manipulation while moving objects. 

Key considerations in our hand design include the following:

- The robotic hand is equipped with force sensors to gather information about contacts with manipulated objects. No other feedback source, such as visual information, is utilized.
- A two-fingered design, with each finger having two joints, is employed for manipulation. This design mirrors a human grasp using the thumb and index fingers, with fingertip movements confined to a plane. Despite this limitation, the design offers a wide range of motion and dexterity, comparable to the human hand’s ability to grasp objects from various orientations and positions.
- Manipulated objects are chosen with varying stiffness and shapes, demonstrating the design’s ability to handle a diverse range of objects, including delicate and rigid ones.
- Aluminum and polylactic acid (PLA) were chosen as the primary materials for manufacturing fingers and related parts to ensure that the hand is lightweight, facilitating swift movements and preventing excessive strain on the robot’s actuators.
- Electronic servomotors are selected as actuators, meeting several design requirements. These motors are equipped with encoders to provide accurate angular position feedback for precise finger positioning. This servo-based gripper enables a fine level of force and speed control, accommodating diverse tasks with variable parameters. Moreover, the use of servo-based grippers contributes to efficient power usage, a crucial factor in extended operation and increased autonomy.

## 3. Design of the Robotic Hand

### 3.1. Prototype Design

**Figure 1:**
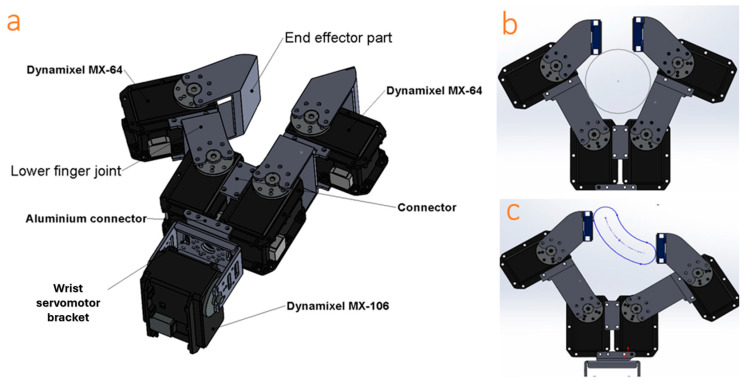
(**a**) A 3D model of the hand design featuring servomotors for actuation, highlighting the end-effector position and incorporating a combination of aluminum and 3D-printed parts. The diverse range of hand configurations enabled it to grip both (**b**) symmetric and (**c**) asymmetric objects.

Figure 1 illustrates the geometric model of our hand module. In designing this module, our objective was to work with and manipulate a variety of objects effectively. Our designed hand comprises two fingers that emulate the functions of the thumb and index fingers. Each finger is equipped with two high-precision servomotors with an impressive accuracy of 4096 resolution by 0.088 degrees. Servomotors are a type of mechanical rotary actuators renowned for their ability to offer precise control over angular position, speed, and torque. These servomotors were positioned at the base and middle segments of each finger to replicate the metacarpophalangeal joint (MCP) at the finger’s base and the proximal interphalangeal joint (PIP) at the middle. Additionally, a single servomotor was employed in the hand module to simulate wrist flexion and extension. We used Dynamixel MX64 servomotors for the finger actuators and one Dynamixel MX106 for the wrist. Dynamixel servomotors are recognized for their intelligence, offering high precision and an impressive torque-to-weight ratio; they are complemented by negligible backlash, gearboxes, and encoders, ensuring smooth and accurate movements. These qualities make them exceptionally suitable for applications that demand both strength and precision in motion. Each of these servomotors can produce a maximum torque of approximately 6 N.m in 12 volts and 4.1 amperes and are designed for full 360-degree rotation, allowing for precise control of angular position. This wide range of motion in each finger joint enables a diverse range of hand configurations for grasping various objects, including symmetric and asymmetric objects. 

Each fingertip of the robotic hand was equipped with a force-sensitive resistor (FSR) pressure sensor, a key component in our grasp control system. These sensors are composed of a polymer resistance material that exhibits a decrease in electrical resistance when subjected to applied pressure or force on their surfaces. Specifically, we utilized FSR 400 sensors featuring a circular active sensing area with a diameter of 12.7 mm. These sensors provide a broad range of resistance values, spanning from tens of ohms in the high-force state to several megaohms in the low-force state, corresponding to a force range of 0–100 N. One notable advantage of these FSR 400 sensors is their rapid response time, allowing our robotic fingers to react quickly to changes in force. To enhance grip and tactile interaction, the entire sensing surface of each sensor was strategically positioned at the center of the fingertips and coated with a layer of silicone rubber. This silicone layer contributes to compliance and provides friction for effective grasping. In our control system, an Arduino board was the interface for reading and processing data from the FSR sensors. The Arduino processed this information in real-time and subsequently transmitted it to the Python programming environment, where our gripper control algorithms were executed. Leveraging the data collected from the FSR sensors, our control software developed in Python (version 3.9) precisely orchestrated commands to the servomotors, enabling the robot to perform comprehensive and reliable grasping operations.

As illustrated in gray in Figure 1a, several 3D-printed PLA and aluminum parts were designed for secure attachment around the two fingers and connections to the actuators. These frames were meticulously designed with precisely sized cavities and channels to accommodate the mounting and wiring of numerous sensors and actuators. The entire modeling and design process of the mechanism and hand module was carried out using the SolidWorks (version 2019) software.

### 3.2. Object and Hand Geometrical Configurations

To determine the appropriate lengths of the robotic hand links, the degrees of freedom at the joints, and the optimized configuration for effective grasping and object manipulation, it is essential to consider the intended functions of the hand and the nature of the objects it will handle. The primary objectives of this hand are to securely grasp objects without causing damage and to manipulate them without the risk of slippage. Achieving these goals necessitates a comprehensive understanding of the size and stiffness characteristics of the objects to be handled.

**Figure 2:**
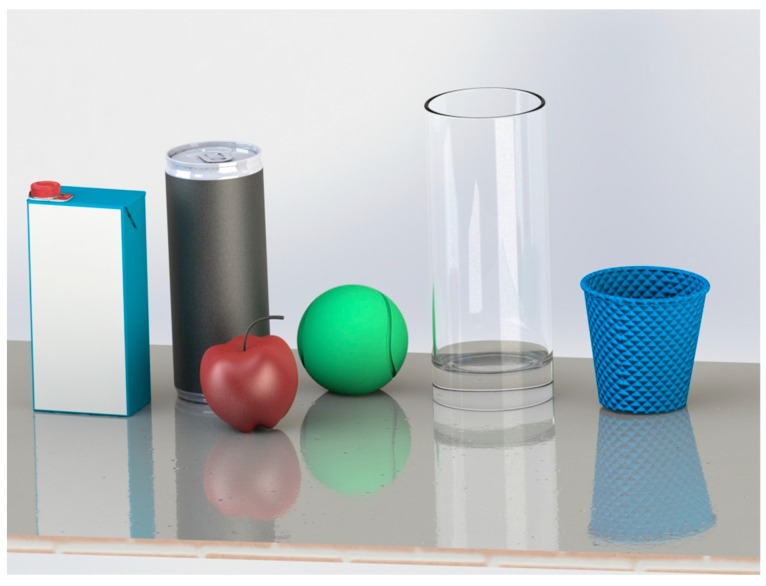
The experimental test objects included a foam ball, an empty soda can, an apple, a glass cup, a plastic cup, and a small milk packet.

In this study, a selection of everyday objects was chosen from the Yale–CMU–Berkeley (YCB) object set, a collection designed to benchmark robotic manipulation capabilities. These objects were carefully chosen to encompass a diverse range of sizes, shapes, and stiffness levels. The target objects for the gripping operations include a sponge ball, an empty soda can, an apple, a glass cup, a plastic cup, and a small milk packet. Visual representations of these objects can be found in Figure 2.

**Figure 3:**
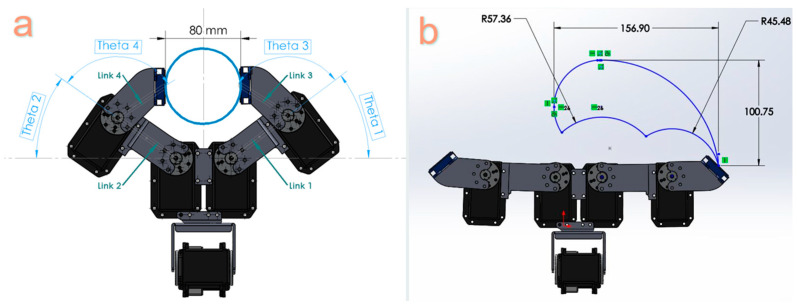
(**a**) Maximum distance between two end effectors when gripping objects. (**b**) The operational area of the robotic hand considering the joint constraints imposed on the servomotors, as outlined in Table 1.

Upon measurement, it was ascertained that all objects had effective diameters falling within the 5-to-7 cm range. Therefore, the initial critical consideration in the design process is to ensure that the robot’s gripper jaws can accommodate these six objects effectively. In simpler terms, the length of the fabricated links (*l*) and the motor rotation range (*θ*) must allow the robot to grasp these objects. The findings of the preliminary experiments are presented in Table 1, which outlines the final link lengths and the range of rotation for the servomotors. These specifications resulted in an aperture that can expand to a maximum width of 8 cm, as demonstrated in Figure 3a. 

Utilizing these specified lengths and ranges of motion, we depicted the operational area for the robot’s right end effector in Figure 3b, as denoted by the blue diagram. Notably, the working space for the left end effector is an exact mirror image of the right finger. Figure 3b also visualizes the overall hand configuration when all servomotors are set to zero.

In addition to establishing the initial hand configuration, achieving the correct end-effector positioning for the secure grasping of each item without risk of slippage or object damage was a pivotal aspect of the hand’s design. To ascertain this optimal configuration, a series of grasping tests was conducted for each of the six objects. These tests aimed to identify the precise end-effector position and distance (fingertip-to-fingertip distance) needed to securely grasp each object. Table 2 provides an overview of the effective diameter of each object and the corresponding required fingertip distance to ensure a secure and damage-free grasp.

The rigidity percentage was calculated by dividing the fingertip-to-fingertip distance by the effective diameter. This ratio was employed to estimate the objects’ stiffness. Using this parameter, the objects were categorized from highest stiffness to lowest stiffness as follows: glass cup, apple, plastic cup, milk packet, empty soda can, and sponge ball.

### 3.3. Joint Controlling Using Inverse Kinematic Equations 

The position of the end effectors, which represent the endpoints of the kinematic chain, were calculated using kinematic and inverse kinematic equations. 

Based on the Denavit–Hartenberg (DH) convention, the transformation matrix between the end effector’s position and wrist position can be expressed as

(1) T=WBT30=T10 T21 T32

(2) T=WBC1C23−C1S23S1L1C1+L2C1C2S1C23−S1S23−C1L1S1+L2S1C2S23C230L2S20001

where “*C*” and “*S*” stand for cosine and sine, respectively. 

By equating the transformation matrices obtained from the forward kinematic equation and the inverse kinematic equation, as they were also used in our previous work [22], the joint angles were determined as follows:

(3) $$\theta_{1}=atan2\left(R_{13}\;,\;{-R}_{23}\right)\;\;\;\theta_{2}=atan2\left(\frac{P_{z}}{L_{2}},C_{2}\right)\;\theta_{3}=atan2\left(\;R_{31}\;,\;R_{32}\;\right)-\theta_{2}$$

### 3.4. Controller Design

There are several different types of robotic controller algorithms, each suited to specific tasks and applications. Some common types of robotic controller algorithms are as follows:

- Position Control: This control algorithm involves controlling the position of the robotic joints to achieve the desired configuration of the end-effectors [23].
- Force Control: This control algorithm involves controlling the forces at the robotic hand’s end effector and is useful for tasks requiring gentle interaction with the environment or objects [24].
- Impedance Control: This control algorithm regulates the mechanical impedance of the end effectors, which involves managing the responses of end effectors to the applied forces and motions. This technique adjusts the resistance of the system to external forces during interactions with objects or the environment [25,26].

The selection of a control strategy depends on factors such as task complexity and performance demands. In this study, given the robot’s intended interaction with various unfamiliar objects, a novel impedance control algorithm was devised to enhance the robot’s adaptability in diverse environments. This adaptability is key to handling a range of tasks, particularly when dealing with soft or deformable materials. It helps the robot to be as rigid or as flexible as needed—firm in free space for precise movements and gentle when interacting with delicate objects to avoid damage. This control algorithm was implemented to improve the robot’s compliance and facilitate smoother motion and interactions by simultaneously controlling velocity and force.

**Figure 4:**
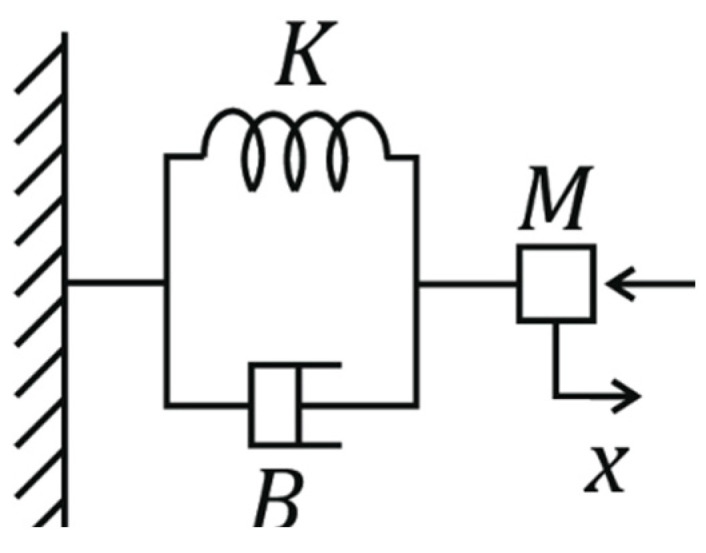
Free body diagram illustrating the dynamics of a mass–spring–damper system.

A mass–spring–damper system, as shown in Figure 4, was used to simulate impedance control because it provides a simplified yet effective model for implementing compliant behavior in robotic systems by adjusting their stiffness and damping properties. The system’s mathematical model is expressed as

(4) $$Z\left(s\right)=\frac{F(s)}{V(s)}=\frac{K}{s}+B+Ms$$

where $Z\left(s\right)$ represents the system’s impedance in the Laplace domain; F(s) and V(s) are the Laplace transforms of force and velocity, respectively; and K, B, and M symbolize the system’s stiffness, damping, and mass parameters. Equation (5) describes the motion of a system consisting of a mass matrix, M(q), attached to a spring with a spring constant matrix, K(q), and a damper with a damping coefficient matrix, B(q), when subjected to an external force, $F\left(t\right)$, as shown in Figure 4. q represents the joint angular position.

(5) $${M\left(q\right).{\ddot{x}}_{e}+B\left(q\right).{\dot{x}}_{e}+K\left(q\right).x}_{e}=F\left(t\right)$$

In this equation, $x_{e}$ is defined as $x_{e}=x_{d}-x$, representing the discrepancy between the desired position, $x_{d}$, and the current position, x, of the end effectors. 

The system stiffness, damping coefficient, and mass were chosen for the system as follows:

Lower stiffness was chosen for softer objects to allow the robotic hand to deform and conform to the object’s shape without applying excessive force. For stiffer objects, higher stiffness was preferable to provide better control, stability, and precision during the grasp.

A lower mass was often desirable for manipulating the soft objects to allow for gentler interactions, which prevents damaging the object. For stiffer objects, a moderate to high mass was chosen to provide higher resistance against deformation. However, it was essential to find a balance to avoid excessive force.

A moderate damping coefficient was optimal for soft objects, facilitating smooth and controlled movements that prevent object deformation. For stiffer objects, a higher damping coefficient allowed the system to quickly dissipate energy and minimize oscillations. The higher damping coefficient was crucial for enhancing the system stability and maintaining control during precise or firm gripping tasks.

The performance of the controller algorithm throughout each step of the gripping task is as follows:

- Approach: The robot starts with a relatively low stiffness and approaches the object. Lower stiffness allows for compliant interaction during the initial contact.
- Contact: As the robot contacts the object, it may gradually increase its stiffness to ensure stability during the grasp. This prevents the excessive deformation of the object.
- Holding: Once the object is securely grasped, the robot can maintain a stable grip by adjusting the stiffness and damping. The control system may continuously adapt to the object’s properties and environmental conditions.
- Lifting: If the task involves lifting the object, the robot can adjust the stiffness and damping to ensure a smooth and controlled lift, minimizing the risk of dropping the object.

Throughout all these steps, the impedance controller adeptly coordinated the movements of the four servomotors, enabling the hand to emulate natural, human-like motions while continuously adjusting the stiffness and damping characteristics of the robotic fingers. In this controller design, the current values measured at each servomotor were used to estimate the external forces acting on the hand. Unlike voltage, which is more commonly used in controller systems, current provides a more precise and direct means of controlling the torque and force output of the servomotors. The integration of current feedback into the impedance control system enhanced the system’s performance by offering more precise insights into the external forces encountered by the robotic hand. 

**Figure 5:**
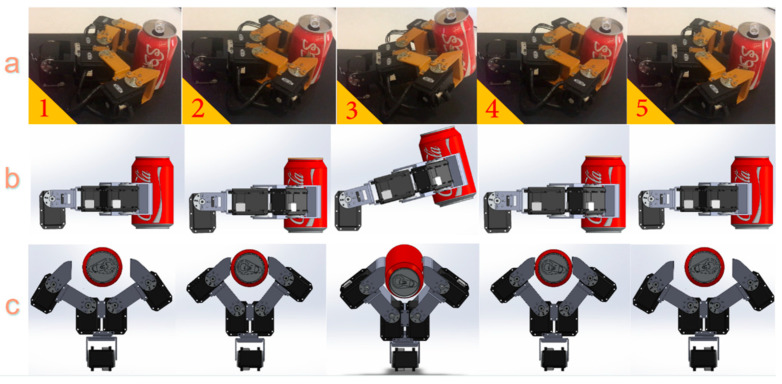
An illustration of (**a**) a successful gripping procedure involving five steps: Step 1 shows the fingers approaching the object. Step 2 demonstrates the fingers making contact with the object and holding it. Step 3 displays the hand lifting the object. Step 4 exhibits the hand setting the object down, and Step 5 portrays the fingers releasing the object. The images in (**b**,**c**) represent the side view and top view of each step, respectively.

The robotic hand had a sense of touch using FSRs integrated into the fingertips. After categorizing objects based on differences in the servomotors’ current, the hand continuously monitored its current and calibrated it, if necessary, to control its grip based on the object’s characteristics, such as stiffness. For example, when grasping a soft object like a milk packet, the required current may vary as the hand adjusts to stabilize the grip. By continuously monitoring, the hand was able to regulate the necessary force. Figure 5 illustrates the five stages of a complete grasping procedure.

## 4. Experimental Implementation

Before implementing the controller, we conducted manual grasping experiments on all six objects to establish the grip force threshold for each. FSR sensors on each fingertip measured the force applied by the fingertips to securely hold each object. A series of experiments yielded a relationship between force and resistance for the FSR sensors. Based on the applied fingertip force measured for each of the six objects, we conducted stiffness categorization, spanning very soft to very stiff, and assigned each object to one of these five distinct categories. Notably, our selection of objects from the YCB object set was deliberate, ensuring that each of the five stiffness categories was represented by at least one object. Table 3 represents the object classification based on the forces recorded by the FSR.

In the manual gripping tests, not only were the applied forces assessed using the FSR, but the servomotor angles for each joint were also meticulously recorded ($\theta_{desired}$). These angle measurements served a dual purpose: a crucial parameter of our controller observer and an additional insight into the kinematics of the robotic hand during the gripping process. Finally, for each class of objects, categorized based on their stiffness and FSR outputs, a force threshold and motor angle were obtained.

After performing the initial manual tests, the actual automated gripping tests involving the controller mechanism and current measurements were performed. These gripping tests were conducted in two separate phases. The first phase was called the *close* phase. This procedure began with the hands transitioning from an open state, with no contact with the objects, to the point where both fingertips made initial contact with objects. Before the closing phase began, we ensured that each fingertip was on each side of an object. This phase finished with the hand being stopped as soon as a sense of touch was detected. This sense of touch was identified by comparing the force at the fingertips with a contact force threshold previously measured using the FSR for our categorization system. In scenarios where the object is not perfectly centered within the gripper, the force calculated from each fingertip may vary. To address this, we ensured that the smaller value was compared with the contact threshold force from the FSRs.

(6) $$F_{g,min}=\min\left(F_{L},F_{R}\right)$$

where $F_{L}$ and $F_{R}$ are calculated forces from FSRs for the left and right fingertips, respectively. This approach empowered the robot to make decisions, ensuring effective operation even when the object was not perfectly centered within the gripper. It is important to note that, before comparing the force values from the FSRs and motor currents, several experiments were conducted to relate the force from the FSRs to Newtons, revealing that ~200 g from an FSR (associated with the softest object in our YCB set) could be equated to ~0.5 N. Thus, as soon as the condition $F_{g,min}>F_{limit\;}=0.5\;N$ was satisfied, the hands paused, and the closing phase was completed with a full touch sensation. FSRs were used at this stage due to their detection speed, which was especially required for detecting the initial contact.

The subsequent phase in grasping objects was the *load* phase, commencing a few milliseconds after the end of the *close* phase (initial contact with the object and full touch sensation). The goal of this phase was to apply an appropriate load to the object for lifting. During this phase, the hand closed an additional 1 mm on each fingertip to ensure that it securely held the object. The current in each of the four servomotors at each joint was measured at this stage and averaged across the two servomotors at each finger. The difference between the current measurement for fingers at the *load* phase ($I_{lL}$ and $I_{lR}$) and the current measurement for fingers at the *close* phase ($I_{cL}$ and $I_{cR}$) was then calculated. A new classification of objects based on their stiffness was developed based on the current differences in these two phases. Table 4 presents the classification of objects based on the current differences in these two phases.

When the hand determined the stiffness class of the object based on the current measurements, the gripping procedure entered its third phase: *load adjustment*. In this phase, based on the degree of stiffness, the servomotors finetuned their angles to apply the necessary load for securely holding objects. For each object, the servomotors adjusted their angles to attain the forces outlined in Table 3, derived from manual experiments across different object classes. This phase also involved minor adjustments in fingertip positions from the loading stage to ensure a more secure and reliable grip. To validate the success of the grasping process, servomotor angles were employed as independent observers. During the load adjustment stage, the servomotor angles ($\theta_{real}$) were measured for each finger when the fingertip applied the appropriate loading. These measured values were then compared with the angles ($\theta_{desired}$) recorded during manual object gripping experiments for each object. Specifically, $\theta_{desired}$ represents the expected servomotor configuration when the distance between two fingertips equals the fingertip-to-fingertip distance for each object, as detailed in Table 2. $\theta_{real}$ denotes the actual angle of the servomotor during the load adjustment cycle obtained in the load adjustment phase. The difference between these two angles is represented as α=|$\theta_{a}-\theta_{m}|$. Through multiple experiments, we consistently observed that α falls within the range of 0 to 2. To achieve more precise adjustments during this phase, allowing for further fingertip loading and positioning accuracy, a target angle was calculated based on the following algorithm.

(7) $$If\;\alpha <1\to Target\;position=(\theta_{real}+\theta_{des})/2$$

(8) $$If\;\alpha >1\to Target\;position=(\theta_{real}+\theta_{des})/2+(0.5\to \theta_{real})$$

Through these adjustments in force and positioning, the hand was carefully maneuvered into the final configuration, which was considered a secure and reliable configuration for gripping objects.

## 5. Results

In this study, our primary objective was to explore the reliability of our control mechanism in the classification of objects based on their stiffness. The controller’s ability to accurately identify objects’ stiffness is of paramount importance, as it enables the robot to apply suitable loading and positioning configurations, ensuring the safe grasping, and lifting of various objects.

As previously mentioned, our initial approach involved manually gripping objects to assess their stiffness based on load values and subsequently classifying them according to their stiffness characteristics. If the classification derived from the current measurements aligns with the FSR-based classification we established earlier, it would validate the effectiveness of our control mechanism. This alignment would further ensure that the appropriate loading is applied so that the robot can grasp the objects securely.

**Figure 6:**
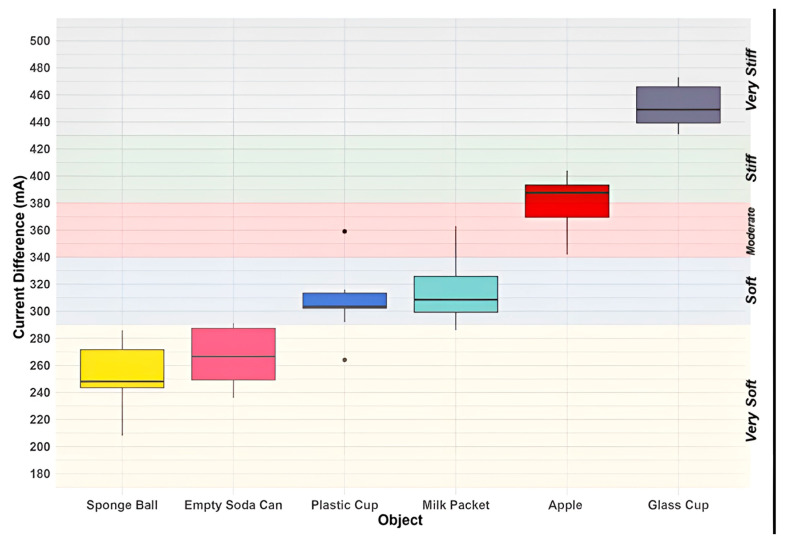
The current difference values for each object measured across ten complete grasping experiments.

To evaluate the robustness of our control techniques in object classification, we conducted ten comprehensive grasping experiments for each of the six objects. During these experiments, we recorded current values for the touching and grasping operations at the end of the close and load phases, respectively. The difference between these current values was employed to categorize the objects according to the criteria outlined in Table 4. The outcomes of these experiments are illustrated in Figure 6 and Table 5. Figure 6 presents box plots for each object, depicting the distribution of current difference values recorded during the ten experiments and illustrating how each object aligns with various stiffness classes based on current differences. Complementing this visual representation, Table 5 summarizes the number of experiments out of ten, where each object has been categorized into different stiffness classes.

Interpreting the obtained results, the sponge ball and empty soda can exhibit characteristics indicative of very soft objects; the plastic cup and milk packet fall into the category of soft objects; the apple and glass cup can be classified as stiff and very stiff objects, respectively.

Following the categorization of objects based on the observed current differences, the robotic hand needed to apply an appropriate force corresponding to the stiffness group identified during manual gripping operations, as detailed in Table 6. The controller sent calculated equivalent current derived from the fingertips’ forces and the associated angles observed by the system to the respective servomotors. This action directed the hand to move to its final configuration.

During 60 experiments, encompassing touching, gripping, and lifting, all objects were handled securely, with no instances of crushing, damage, or slippage recorded. The resultant loading and positional configurations for each object were averaged through ten experiments and are presented in Table 6, providing a comprehensive overview of the safe and effective manipulation achieved through the control system. 

## 6. Summary and Conclusions

This paper aimed to design, prototype, and evaluate a novel robotic hand augmented with an inexpensive haptic mechanism capable of lifting unknown objects. To this end, we successfully fused FSRs to improve speed and servomotor current to improve accuracy in grasping objects. FSR sensors granted the robotic hand a basic touch sensitivity akin to the human hand’s initial contact with an object. The control strategy then refined the grip by adjusting the servomotors’ force based on current measurements, marrying delicate touch with precise force control. Six predetermined objects were selected for evaluation; however, the robot has the ability to grasp new objects. Our designed hand showed a high level of accuracy and skill compared with previously proposed designs. To prototype the mechanical structure of the proposed hand, the griping aperture limit (considering the dimensions of the test objects) and the workspace of each finger were examined.

For the mechatronic design of the robot, a communication protocol between the motors and the processor was established, and two parallel procedures were employed to control the robot: first, touching the object, which was detected through the fingertips’ FSRs and commanded the motors to stop; second, lifting the object by monitoring the motor currents and the impedance control. Inverse kinematic equations were used as observers for the control system. Our results demonstrated the following:

- The use of servomotors in the design of the hand or gripper increased accuracy and ease of operation, enabling the implementation of control algorithms.
- Two degrees of freedom in each finger enhanced the robot’s dexterity.
- Using precise servomotors allowed for the application of direct and inverse kinematics to control the robot’s position as a controller or observer.
- Impedance control allowed for an acceptable understanding of touching different objects and control of the hand, providing simplicity and high precision.

In the future, we will use this design to grasp an object using all connected linkages. Each link will not move after touching the object. To improve grasping, we will design a control algorithm that prevents the slippage of objects. For example, by reading the current at each end effector and employing multiple force sensors at the fingertips, the robot can profile the slippage trajectory and intervene accordingly. Furthermore, designing an arm robot using our proposed hand as an end effector or combining the hand with an industrial arm to relocate objects could be interesting. 

Developing a robotic hand that can match the dexterity of a human hand is still a distant reality. However, it is crucial to prioritize human-robot interactions and intuitive control when designing such a robotic hand to ensure high user compliance. We need to reduce the cost of our design and improve the range of objects it can handle. Currently, the design is limited to the servomotors that provide the current feedback. Nevertheless, we have made significant progress toward achieving this goal.

## Figures and Tables

**Table 1:** The length of links and servo rotation range are specified for each joint.

Links	Length (mm)	Joints	Joint Constraints (Degree)
Link 1	67 mm	Servo 1	0 < *θ*_1_ < 90
Link 2	67 mm	Servo 2	90 < *θ*_2_ < 180
Link 3	40.3 mm	Servo 3	0 < *θ*_3_ < 110
Link 4	40.3 mm	Servo 4	70 < *θ*_4_ < 180

**Table 2:** Results of manual gripping experiments: effective diameter refers to the diameter of each object before gripping; fingertip-to-fingertip diameter refers to the diameter of each object after gripping; and the rigidity percentage represents the ratio of an object’s diameter after gripping to its diameter before gripping.

Objects	Effective Diameter (mm)	Fingertip-to-Fingertip Distance (mm)	Rigidity Percentage (%)
Sponge Ball	65	58	89
Empty Soda Can	50	47	94
Apple	60	60	100
Glass Cup	52	52	100
Plastic Cup	60	59	98
Milk Packet	42	40	95

**Table 3:** Stiffness classification of objects based on the FSR values.

Degrees of Stiffness	Values from FSR Sensors
Very Soft	0–200
Soft	201–400
Moderate	401–550
Stiff	551–700
Very Stiff	701–800

**Table 4:** Stiffness classification of objects based on the current differences in the *close* and *load* phases.

Degrees of Stiffness	Current Differences in Close and Load Phases (mA)
Very Soft	80–289
Soft	290–339
Moderate	340–379
Stiff	380–429
Very Stiff	≥430

**Table 5:** The number of times each object fell into a stiffness category out of ten grasping trials.

Objects	Very Soft	Soft	Moderate	Stiff	Very Stiff
Sponge Ball	10	0	0	0	0
Empty Soda Can	9	1	0	0	0
Plastic Cup	1	8	1	0	0
Milk Packet	1	7	2	0	0
Apple	0	0	3	7	0
Glass Cup	0	0	0	0	10

**Table 6:** A summary of grasping experiment results for different objects.

Experimental Results	Sponge Ball	Empty Soda Can	Plastic Cup	Milk Packet	Apple	Glass Cup
Average servomotor current values during initial touch (end of close phase)	157.7 mA	142.7 mA	167.4 mA	155 mA	167.2 mA	163.5 mA
Average servomotor current values during loading (end of load phase)	319.2 mA	445 mA	469.9 mA	472 mA	571.5 mA	600.2 mA
The current difference at the close and load phases	161.5 mA	282.2 mA	302.5 mA	317 mA	404.2 mA	436.7 mA
Degrees of stiffness	Very Soft	Very Soft	Soft	Soft	Stiff	Very Stiff
The position of left and right fingertips during touch (x,y)	(166, 40) and (166, −40)	(166, 40) and (166, −40)	(166, 35) and (166, −35)	(170, 19) and (170, −19)	(166, 34) and (166, −34)	(166, 34) and (166, −34)
The position of left and right fingertips during gripping (x,y)	(175, 25) and (175, −25)	(171, 29) and (171, −29)	168, 32) and (168, −32)	(175, 14) and (175, −14)	(169, 31) and (169, −31)	(167, 33) and (167, −33)
The position of left and right fingertips during destructive deformation (x,y)	NA	(176, 24) and (176, −24)	(170, 31) and (176, −31)	(180, 9) and (180, −9)	NA	NA

## Data Availability

The data will be available on GitHub.
